# Supplementary figures and images for: The Microtubule-Associated Innate Immune Sensor GEF-H1 Does Not Influence Mouse Norovirus Replication in Murine Macrophages
Source: Viruses. 2019 Jan 10;11(1):47. doi: 10.3390/v11010047 (PMC6356845; doi:10.3390/v11010047)

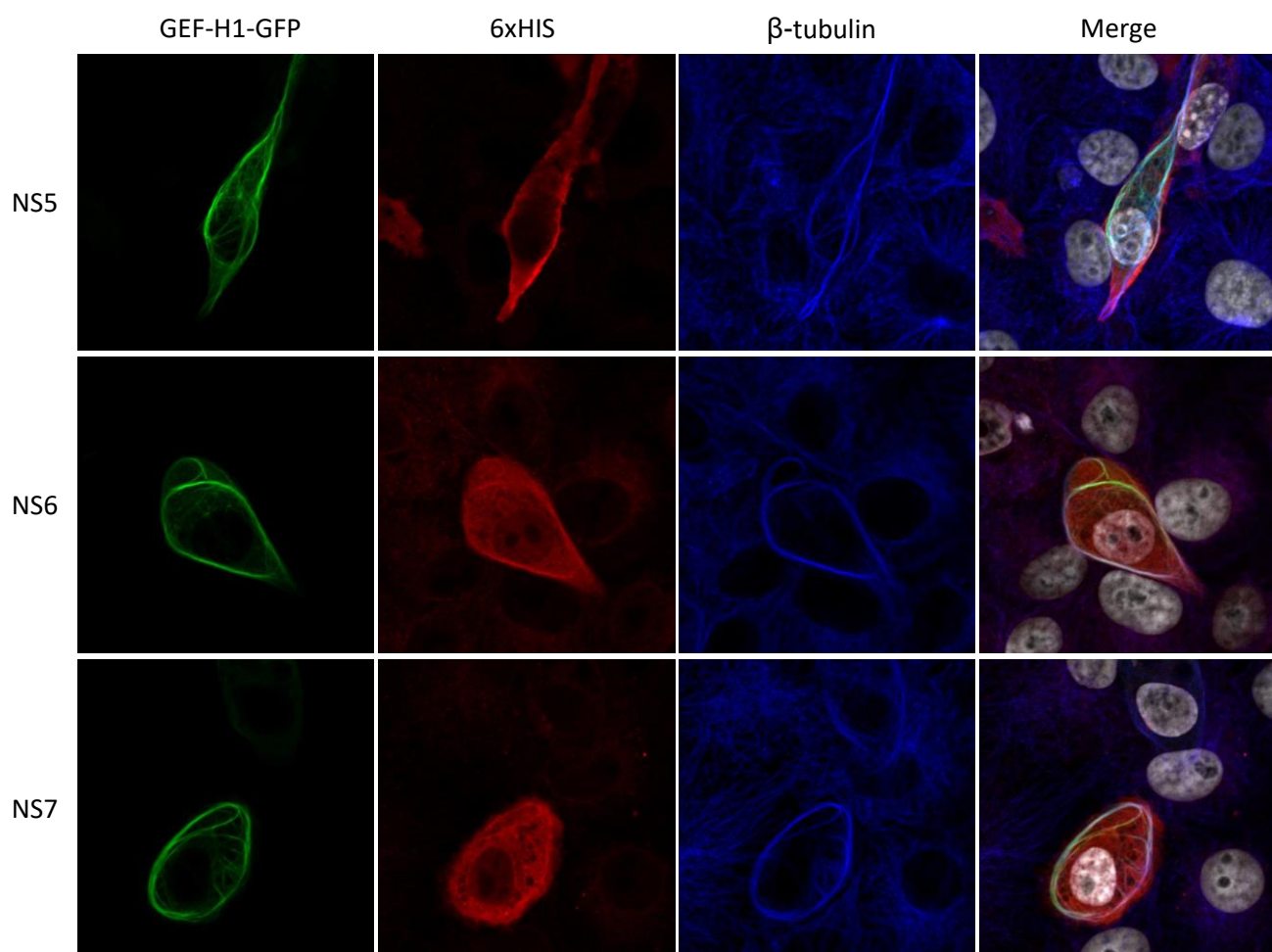

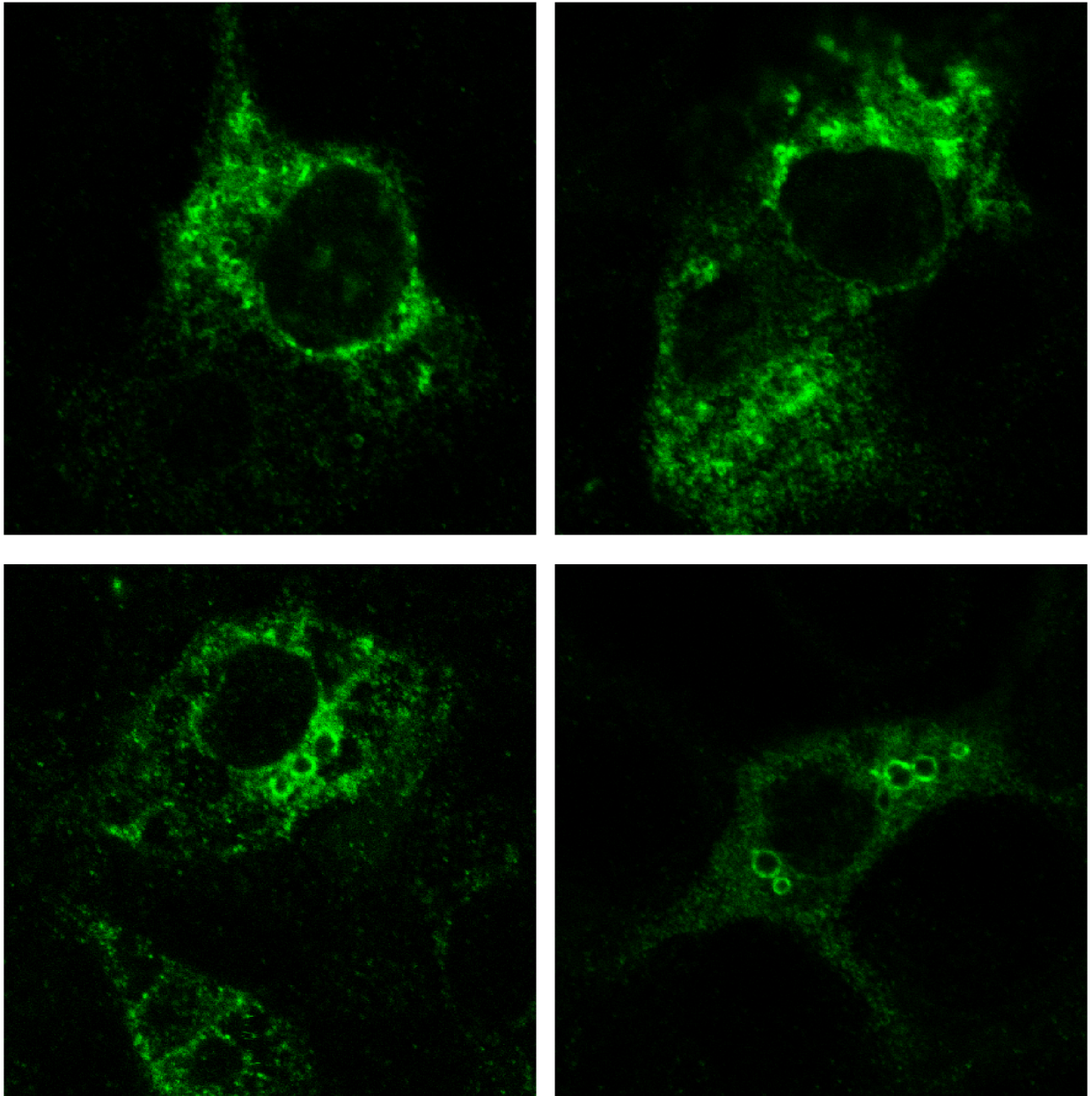

Supplementary Figure 2  
Fritzlär, White and Mackenzie

Supplement: Supplementary file 1 [file viruses-11-00047-s001.pdf]
